# Acute Kidney Injury, Recurrent Seizures, and Thrombocytopenia in a Young Patient with Lupus Nephritis: A Diagnostic Dilemma

**DOI:** 10.1155/2016/7104098

**Published:** 2016-12-04

**Authors:** Hector Alvarado Verduzco, Anjali Acharya

**Affiliations:** ^1^Department of Internal Medicine, Division of Internal Medicine, Jacobi Medical Center, Bronx, NY, USA; ^2^Department of Nephrology, Division of Internal Medicine, Jacobi Medical Center, Bronx, NY, USA

## Abstract

*Introduction*. Posterior reversible encephalopathy syndrome (PRES) is a constellation of clinical and radiologic findings. Fluctuations in blood pressure, seizures, and reversible brain MRI findings mainly in posterior cerebral white matter are the main manifestations. PRES has been associated with multiple conditions such as autoimmune disorders, pregnancy, organ transplant, and thrombotic microangiopathy (TMA).* Case Presentation*. A 22-year-old woman with history of Systemic Lupus Erythematous complicated with chronic kidney disease secondary to lupus nephritis class IV presented with recurrent seizures and uncontrolled hypertension. She was found to have acute kidney injury and thrombocytopenia. Repeat kidney biopsy showed diffuse endocapillary and extracapillary proliferative and membranous lupus nephritis (ISN-RPS class IV-G+V) and endothelial swelling secondary to severe hypertension but no evidence of TMA. Brain MRI showed reversible left frontal and parietal lesions that resolved after controlling the blood pressure, making PRES the diagnosis.* Conclusion*. PRES is an important entity that must be recognized and treated early due to the potential reversibility in the early stages. Physicians must have high suspicion for these unusual presentations. We present a case where performing kidney biopsy clinched the diagnosis in our patient with multiple confounding factors.

## 1. Introduction

Posterior reversible encephalopathy syndrome (PRES) was initially described by Hinchey et al. in 1996 in a retrospective series of 15 patients with headache, seizures, altered mental status, high blood pressure, and unique MRI findings that disappeared on subsequent imaging after controlling blood pressure [1].

The exact incidence of PRES is unknown, commonly being mistaken for acute stroke. Some authors have reported in their case series predominance in young female patients (75%) particularly when being associated with autoimmune disorders, such as Systemic Lupus Erythematous (SLE) with nephritis [2–4]; but it can be present at any age or gender. PRES can be a challenging diagnosis especially when multiple comorbidities are present, as illustrated in our patient, masking the diagnosis and delaying the proper treatment. We propose an algorithm in the diagnosis of PRES associated with autoimmune disorders and acute kidney injury.

## 2. Case Report

A 22-year-old woman with history of Systemic Lupus Erythematous (SLE) complicated with chronic kidney disease (CKD) secondary to lupus nephritis [ISN-RPS class IV-G (A)] presented with generalized tonic-clonic seizures and uncontrolled hypertension. Vitals signs at presentation were as follows: blood pressure of 180/148 mmHg; heat rate of 140 per minute. She was afebrile and somnolent but responsive to verbal stimuli and oriented in time, place, and person with no evidence of apparent focal deficits. She had no body rashes, ulcers, hair loss, joint tenderness, or swelling. The rest of the physical examination was unremarkable.

Laboratory tests on presentation were remarkable for anemia, thrombocytopenia, elevated lactate dehydrogenase, and a reticulocyte count of 3.3% (Table 1). Urine toxicology and pregnancy test were negative. Urinalysis showed protein >300 and no evidence of infection. She received hydrocortisone, lorazepam, and levetiracetam and was loaded with phenytoin.* An initial head CT scan revealed acute left frontal and parietal lobe infarcts*. Patient was admitted to the Intensive Care Unit (ICU) with presumptive diagnosis of acute ischemic stroke versus lupus vasculitis or cerebritis. Peripheral smear revealed marked anisocytosis and 4 to 10 schistocytes per HPF.

She was diagnosed presumptively with thrombotic thrombocytopenic purpura (TTP) due to anemia, thrombocytopenia, and schistocytes on peripheral smear. Plasma exchange and prednisone were started as the mortality rate without early treatment in TTP is very high. Additional testing was pursued to rule out other causes.

Further tests showed antinuclear antibody 1 : 1280 with speckled pattern; antibodies including anti-Smith, RNP, dsDNA, and histone were also positive. Lupus anticoagulant and cardiolipin antibody were negative.

**Figure 1:**
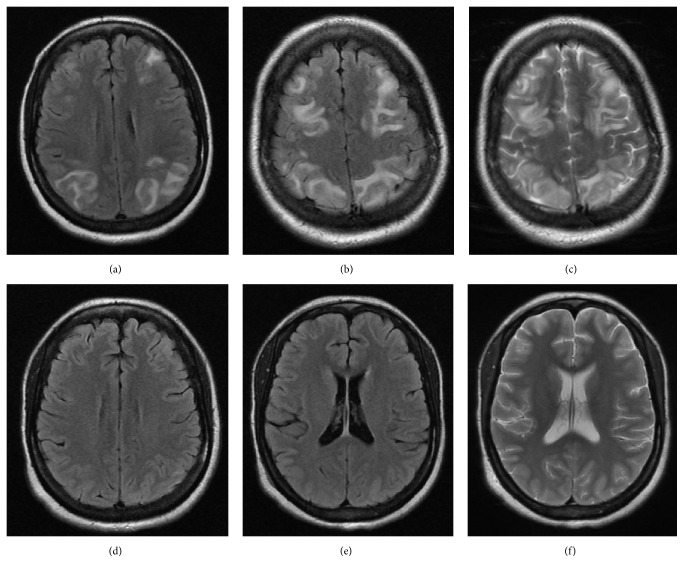
The initial MRI (images (a)–(c)) showed typical holohemispheric involvement: axial view in FLAIR (see (a), (b)) and T2-weighted (see (c)) images with extensive areas of high signal intensity in the subcortical white matter of both parietooccipital regions, including frontal lobes. Second MRI (images (d)–(f)) performed 2 weeks later with no signal abnormality within the brain parenchyma on FLAIR (see (d), (e)) and T2-weighted (see (f)) images.

Electroencephalogram (EEG) was with no epileptiform discharges. First brain MRI showed extensive areas of high signal intensity in the subcortical white matter of both parietooccipital regions, as well as in the frontal lobes and right basal ganglia/capsular region and the head of the caudate nucleus on the left (Figures 1(a)–1(c)) on both FLAIR and T2-weighted images.

**Figure 2:**
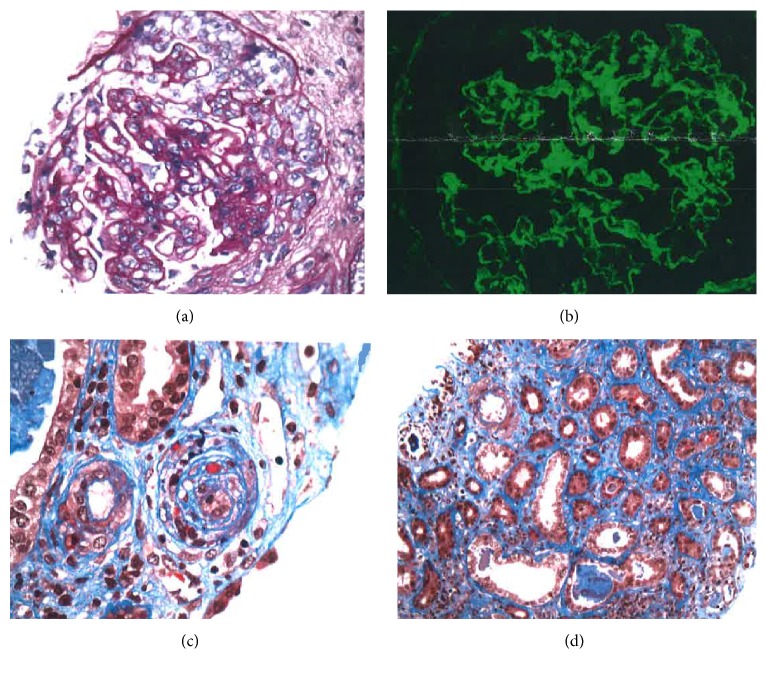
Renal biopsy with crescent formations (image (a)). Immunofluorescent findings (image (b)) of diffuse granular mesangial and glomerular capillary wall staining for IgG, IgM, IgA, C3, and C1; tubular basement membrane and arteriolar staining for IgG, IgM, and C3; vessel wall staining for IgG, IgM, and C3. Arterioles with focal luminal obliteration and concentric intimal fibroplasia (image (c)). Moderate fibrosis (image (d)). Findings consistent with lupus nephritis and severe hypertension.

She completed a ten-day course of plasma exchange but continued to have low platelets, anemia, and worsening kidney function. ADAMTS13 assay level returned back to normal (82%) and also paroxysmal nocturnal hemoglobinuria panel was negative. At this time, after an interdisciplinary team meeting, a decision was made to perform a kidney biopsy. She was considered to have very high risk of the procedure due to persistent thrombocytopenia, anemia, and high risk of bleeding; but this was thought to be essential in order to clarify the diagnoses which at this time were mainly lupus nephritis versus atypical hemolytic uremic syndrome. Nicardipine IV infusion was continued for a more stable and tight blood pressure control. Patient received one dose of complement based therapy with eculizumab one day before the kidney biopsy (day 14 of hospitalization). The kidney biopsy showed diffuse endocapillary and extracapillary proliferative and membranous lupus nephritis (ISN-RPS class IV-G+V), new changes with endothelial swelling secondary to severe hypertension, but no evidence of thrombotic microangiopathy (Figure 2). Eculizumab was stopped. The platelet count and hemoglobin level started to increase and BP was better controlled.

Second MRI of the head was performed weeks later which confirmed reversal of the brain lesions seen on the first MRI (Figures 1(d)–1(f)).

Unfortunately her kidney function did not improve, requiring permanent renal replacement therapy. She required renal replacement therapy for a brief period for optimizing her volume status. Her BP medications were optimized, requiring maximum doses of labetalol, nifedipine, clonidine, and minoxidil. She was transferred to acute rehabilitation service where she had multiple episodes of seizures due to uncontrolled BP secondary to poor medication compliance. Third MRI performed showed diffuse bilateral peripheral cortical and subcortical signal abnormalities including the posterior fossa and cerebellum. She left the hospital without completing rehabilitation and unfortunately was lost to follow-up.

## 3. Discussion

This case raises awareness of PRES in the differential diagnosis of acute stroke. Most of the presentations of PRES are straightforward, but in some patients with confounding factors the diagnosis is not obvious. This is especially true when the patient presents with acute or chronic manifestation of their underlying disease or is on a medication that can predispose to PRES, making the diagnosis challenging.

PRES can occur with variable degrees of hypertension regardless of its etiology. A rapid rise in blood pressure is a greater risk for development of PRES than the degree of hypertension itself [5]. The majority of PRES cases present only with mild increases in blood pressure [6, 7] with average maximum mean arterial blood pressure (MAP) of 160 mmHg and in some cases even with normal blood pressure [8]. Other frequent findings are seizures (60–75%), mostly generalized tonic-clonic type [2, 9–12], altered mental status [11–13], visual disturbances [10, 11, 13, 14], severe headache [12, 14, 15], status epilepticus (5–15%) [2, 10, 12], and nausea and vomiting [6, 7].

Despite a plethora of information on PRES in general, there is a paucity of data in those with established chronic kidney disease (CKD), even though these patients have a high preponderance of risk factors.

*Settings in Which PRES May Be Likely to Develop*

Autoimmune disorders: Systemic Lupus Erythematous, antiphospholipid syndrome, polyarteritis nodosa, cryoglobulinemia, thrombotic thrombocytopenic purpura, scleroderma, polyangiitis, antiglomerular basement membrane antibody disease, rheumatoid arthritis, Sjögren syndrome, Crohn's disease, ulcerative colitis, autoimmune hepatitis, type 1 diabetes mellitus, Grave's disease, Hashimoto thyroiditis, and neuromyelitis optica [2, 16–20]
Essential hypertension
Preeclampsia and eclampsia [21, 22]
Acute or chronic renal failure and dialysis (55%)[23–27]
Septicemia and severe infections (predominantly gram positive organisms) [4, 28]
Immunosuppressive therapy (despite normal levels): cisplatin, cyclosporine, tacrolimus, intravenous globulin, rituximab, methotrexate, bevacizumab, sunitinib, and sorafenib [15, 29–33]
Others: blood transfusion, contrast exposure, hypercalcemia, cocaine, and methamphetamine

The incidence has been variably reported, with a study from Ireland showing an occurrence of 0.84% [34]. Patients with CKD and end stage renal disease (ESRD) have the perfect setup for the development of PRES such as a higher MAP, volume overload, electrolyte abnormalities, and underlying chronic vascular disease. Despite this, the incidence reported seems to be low except in the realm of solid organ transplantation and in those receiving peritoneal dialysis [35–37]. Though originally described as a reversible lesion, the Berlin PRES study showed that 43% had incomplete resolution of edema and this was associated with a higher MAP at presentation [11].

The pathophysiology of PRES is not fully understood and two theories are proposed [16] both with limitations. One theory proposes that severe systemic hypertension leading to hyperperfusion overwhelms the autoregulatory capacity of the cerebral vasculature (principally arterioles) and results in increased capillary pressure and vasogenic edema. According to the second theory, the principal problem is cerebral vasoconstriction that causes downstream hypoperfusion, ischemia, and capillary leak. Blood flow dysregulation is thought to cause blood brain barrier (BBB) disruption and endothelial damage. In addition, drugs and certain underlying disease processes can cause direct endothelial injury [31] and disruption of the blood brain barrier. But in most of the cases it is related to fluctuations of the blood pressure [24] with subsequent disordered cerebral autoregulation and endothelial dysfunction [16], possibly from cytokines [38] and upregulation of vascular endothelial growth factor. The hydrostatic edema is characteristically seen on MRI on FLAIR and T2-weighted images.

PRES was initially described in a case series [1] of diverse diseases including SLE, hypertensive encephalopathy, acute nephritis, eclampsia, melanoma, and multiple posttransplant immunosuppressed patients that were taking cyclosporine [9, 39] or tacrolimus [1, 2, 9]. In patients with SLE, it has been found that aggressive immunosuppressive therapy, high SLEDAI scores, renal dysfunction, and uncontrolled hypertension contribute to the development of PRES [40–42].

Brain imaging is essential in the diagnosis of PRES. Typically seen are asymmetric cortical and subcortical white matter edema in the posterior cerebral hemispheres not confined to a single vascular territory [9]. Particularly affected are the parietooccipital regions (94–98%) and frontal (77%–79%) and temporal (64%–68%) lobes. Less commonly involved areas include the cerebellum (35%), mainly in patients with underlying autoimmune disease, as well as brainstem and basal ganglia (10%) [43, 44]. These changes are classically described as punctuate or confluent areas of T2-hyperintense vasogenic edema on MRI, being more sensitive on fluid-attenuated inversion recovery (FLAIR) [43].

**Figure 3:**
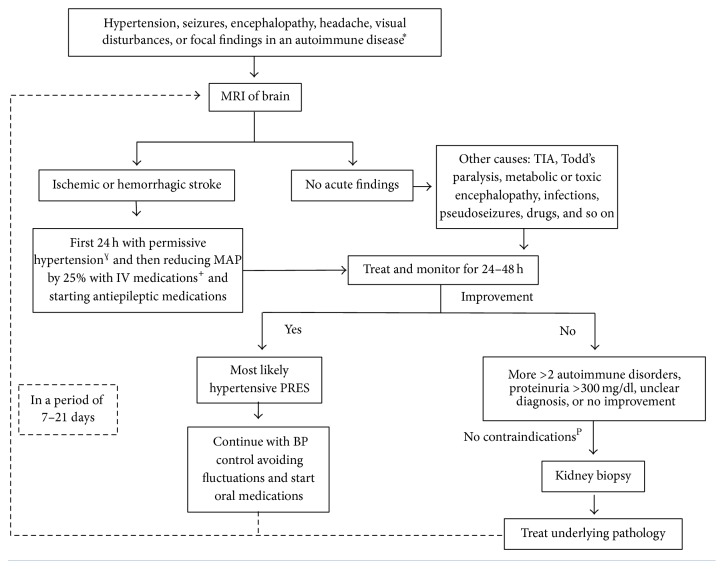
Diagnostic approach for the evaluation of possible PRES in patients with baseline autoimmune disease. It needs more than 2 criteria present for kidney biopsy. MRI: magnetic resonance; MAP: mean arterial blood pressure; PRES: posterior reversible encephalopathy syndrome; BP: blood pressure; TIA: transient ischemic attack. ^*∗*^Systemic Lupus Erythematous, antiphospholipid syndrome, polyarteritis nodosa, cryoglobulinemia, thrombotic thrombocytopenic purpura, scleroderma, granulomatosis with polyangiitis, antiglomerular basement membrane antibody disease, rheumatoid arthritis, Sjögren syndrome, Crohn's disease, ulcerative colitis, autoimmune hepatitis, type 1 diabetes mellitus, Grave's disease, Hashimoto thyroiditis, and neuromyelitis optica. ^+^Preferably, nicardipine, labetalol, nitroprusside, enalaprilat, or hydralazine. ^P^Kidneys <9cm, solitary native kidney, multiple, bilateral cysts, or renal tumor; uncorrectable bleeding diathesis, severe hypertension or hemodynamic instability, hydronephrosis, active renal infection, skin infection over biopsy site, severe anatomic abnormalities, and uncooperative patient. ^*ɣ*^Permissive hypertension defined as follows: no need for BP control unless SBP >220 mmHg, DBP >120 mmHg, and patient has active ischemic coronary disease, heart failure, aortic dissection, acute renal failure, hypertensive encephalopathy, preeclampsia/eclampsia, or indications for thrombolytic therapy (maintaining BP <185/110 mmHg).

It is imperative not only to correctly identify PRES as an entity but also to identify the underlying cause(s) that are triggering this syndrome.* There are no specific diagnostic criteria or algorithm for PRES*. We present a diagnostic algorithm that can be used in these particularly difficult cases with kidney injury (Figure 3). In this algorithm we suggest the crucial role that performing kidney biopsy can play when the cause is not obvious or there are multiple triggers present, so we can properly direct the therapy and reduce complications from treatment [45]. In our case, although it was a difficult decision to make, the kidney biopsy ruled out aHUS and helped guide treatment. In addition, compared to the previous kidney biopsy, there was less histologic activity and more chronicity. The arterioles display focal luminal obliteration, concentric intimal fibroplasia, endothelial cell swelling, and entrapped RBCs, which could be secondary to severe hypertension. With these findings it was possible to stop the administration of eculizumab, reducing costs of treatment tremendously (approximate yearly cost per patient: USD $400,000). Eculizumab is a humanized recombinant immunoglobulin G2/4 monoclonal antibody that has been shown to be effective in the treatment of aHUS by linking to complement protein C5 and preventing the formation of membrane attack complex C5b-9. It was demonstrated in two studies that the use of eculizumab in aHUS reduces endothelial damage and thrombosis of the kidney vasculature [46, 47]; its use was approved by FDA for management of aHUS in 2011 and is currently considered first-line treatment. In adults with aHUS it is administered intravenously 900 mg weekly for 4 doses with a maintenance dose of 1,200 mg at week 5 and 1,200 mg every 2 weeks thereafter.

There is no specific treatment for PRES. Prompt and moderate lowering of blood pressure along with removal of the underlying cause is the mainstay of treatment. Delay in diagnosis and treatment can result in cerebral infarction or hemorrhage with persistent neurologic damage and chronic seizures. There are no established guidelines for BP reduction, but aiming for an initial 20% reduction in MAP is usual [48]. It is important to avoid a precipitous drop in blood pressure. Control of the blood pressure will often be followed by dramatic improvement within days to weeks [2, 10, 15]. The goal is to decrease the mean arterial blood pressure by 25% within the first few hours, preferably with continuous intravenous medications to avoid BP fluctuations [49]. The preferred medications are nicardipine, labetalol, nitroprusside, enalaprilat, or hydralazine [50, 51]. Patients with seizures are preferably treated with phenytoin or other antiepileptic medication depending on the patient's comorbidities [52].

The prognosis is favorable and most patients will recover with prompt treatment [53]. Nevertheless a small percentage (3–6%) will have an unfortunate outcome despite treatment [14, 23, 54, 55] such as intracranial hemorrhage, posterior fossa edema with brainstem compression or hydrocephalus, and diffuse cerebral edema with increased intracranial pressure [56, 57]. Hyperglycemia is an independent factor associated with poor outcome [14]. There are unique adverse prognostic factors for developing PRES-related intracranial hemorrhage (ICH) especially in patients with lupus, including hypoalbuminemia, thrombocytopenia, and SLEDAI score >18 [17]. The recurrence of PRES is rare, reported in 4 to 8% of cases [53, 58]. Most of the recurrences are caused by unsatisfactorily controlled blood pressure [59], occurring between 30 days and 2 years after the initial episode [60, 61].

In our patient, the initial presentation was very complicated and posed a diagnostic and therapeutic dilemma. In addition despite atypical features and presenting with 2 of the adverse prognosticators, there was no* ICH* during the course. The patient also had recurrence which is unusual. The recurrent episodes of seizures correlated with high blood pressure and were confirmed as PRES with MRI. In addition, the case highlights that atypical MRI findings involving cerebellum and basal ganglia may occur.

## 4. Conclusions

Posterior reversible encephalopathy syndrome should be included as a differential diagnosis in any cause of headache, acute confusional state, seizure, and acute stroke. It must be recognized and treated timely because of the potential reversibility in the early stages. Physicians must have high suspicion for unusual presentations. In patients with AKI or CKD as in our patient, having a low threshold for performing kidney biopsy is helpful when the diagnosis is obscure.

## Figures and Tables

**Table 1:** Laboratory data.

Variable	Reference range, adults	On admission	Third hospital day	Sixteenth hospital stay	At discharge
White cells (per mm^3^)	3,500–11,000	29,000	14,500	8,900	8,800
Hemoglobin (g/dl)	12–16 (women)	9.6	7.7	7.7	8.9
Hematocrit (%)	36–46	30.6	23.9	22.4	26.3
Platelets (per mm^3^)	150,000–440,000	95,000	45,000	143,000	131,000
Carbon dioxide (mEq/L)	24–30	12.6	24.8	29.5	21
Urea nitrogen (mg/dl)	5–26	54	45	47	59
Creatinine (mg/dl)	0.1–1.5	2.3	2.2	5.3	2.9
Albumin (g/dL)	3.5–5.5	2.4	2.3	2.5	3.3
Lactate dehydrogenase (U/L)	100–210	1,144	418	393	
Total bilirubin (mg/dl)	0.1–1.2	0.2	0.3	0.3	0.3
Erythrocyte sedimentation rate (mm/hr)	0–20	36			
C-reactive protein (mg/L)	0–5	64.9			
Complement C3 (mg/dl)	90–180	80			
Complement C4 (mg/dl)	10–40	28.5			
ADAMTS13 activity	≥67%	82%			
Direct Coombs test	Negative	Negative		Negative	
